# Supplementary material for: The novel BET‐CBP/p300 dual inhibitor NEO2734 is active in SPOP mutant and wild‐type prostate cancer
Source: EMBO Mol Med. 2019 Sep 26;11(11):e10659. doi: 10.15252/emmm.201910659 (PMC6835201; doi:10.15252/emmm.201910659)
Supplement: Supplementary file 1 — Appendix [file EMMM-11-e10659-s001.pdf]

APPENDIX

Table of Content

Table of content……………………………………………………………………………….1

Appendix Table S1……………………………………………………………………………2

Appendix Table S2……………………………………………………………………………3

Appendix Table S3……………………………………………………………………………3

Appendix Table S4…………………………………………………………………………4 - 5

Appendix Table S1. Primer sequence for Sanger sequencing, RT-qPCR and ChIP-qPCR

Gene	Usage	Forward (5'- 3')	Reverse (5'- 3')
SPOP (Q165P)	Sequencing	TTTGCCCAGGTTTGTAGTCC	TCATCATCCATGCCTTGAAA
SPOP (Exon 6)	Sequencing	ACCCATAGCTTTGGTTTCTTCTCCC	TATCTGTTTTGGACAGGTGTTTGCG
SPOP (Exon 7)	Sequencing	ACTCATCAGATCTGGGAACTGC	AGTTGTGGCTTTGATCTGGTT
GAPDH	RT-qPCR	GAAGGTGAAGGTCGGAGTC	GAAGATGGTGATGGGATTTC
PSA	RT-qPCR	AGGCCTTCCCTGTACACCAA	GTCTTGGCCTGGTCATTTCC
NKX3.1	RT-qPCR	GTACCTGTCGGCCCCTGAAC	GGAGAGCTGCTTTCGCTTAG
TMPRSS2	RT-qPCR	TGGAGGTTGCTCAGGACTTC	GAGTTCAAAGCCATCTTGCTG
RAC1 promoter	ChIP-qPCR	CCAAAGTGTTGGGATTACGG	CGGAGTTTCTCTGGACTTCG
c-MYC enhancer	ChIP-qPCR	GGCTTACAGGATACCCCCAACT	GGGCTATCACACCTCGCCC

Appendix Table S2. Patient-derived xenograft (PDX)

Name	Origin	Mutation status	Usage in this manuscript	Reference
LTL313HR (SPOP WT)	Primary prostate cancer	PTEN loss	Used in xenograft and organoid culture	First reported in this manuscript
LTL573R (SPOP Q165P)	Liver metastases tissue	SPOP mutation (Q165P), PTEN loss	Used in xenograft and organoid culture	First reported in this manuscript
V2-Met (SPOP WT)	Castrate-resistant prostate cancer (CRPC) metastases tissue	SPOP WT, *TMPRSS2-ETV4* fusion	Used in xenograft and organoid culture	(Kohli et al, 2015)

Appendix Table S3. Patient-derived organoids

Name	Origin	Mutation status	Collaboration	Reference
LTL313HR	PDX originally derived from primary prostate cancer	SPOP WT, PTEN loss	BC Cancer Research Centre, Canada	First reported in this manuscript
LTL573R	PDX originally derived from liver metastases tissue	SPOP mutation (Q165P), PTEN loss	BC Cancer Research Centre, Canada	First reported in this manuscript
MSK-PCa1 (BM1)	L2 vertebral body	SPOP WT, PTEN loss	Memorial Sloan Kettering Cancer Center, New York, USA	(Gao et al, 2014; Zhang et al, 2017)
MSK-PCa2 (BM5)	Acetabulum	SPOP WT, PTEN loss	Memorial Sloan Kettering Cancer Center, New York, USA	(Gao et al, 2014; Zhang et al, 2017)
MSK-PCa3 (ST1)	Retroperitoneal lymph node	SPOP WT, PTEN loss	Memorial Sloan Kettering Cancer Center, New York, USA	(Gao et al, 2014; Zhang et al, 2017)
MSK-PCa15 (ASC1)	Primary prostate cancer	SPOP mutation (W131R)	Memorial Sloan Kettering Cancer Center, New York, USA	(Gao et al, 2014; Zhang et al, 2017)

Appendix Table S4. Detailed *P* values and sample number (n)

Figure	Compared groups	Symbol	*P* Value
Fig 1E	SPOP WT (n = 85) vs. SPOP MUT (n = 12)	**	0.0012
Fig 1G	SPOP WT (n = 85) vs. SPOP MUT (n = 12)	***	0.0006
Fig 4B	In lenti-EV groups:<br>DMSO (n = 15) vs. JQ1 (n = 5)<br>DMSO (n = 15) vs. CPI-637 (n = 5)<br>DMSO (n = 15) vs. JQ1 + CPI-637 (n = 5)<br>DMSO (n = 15) vs. NEO2734 (n = 5)	 ***<br>***<br>***<br>***	 1.59E-06<br>8.69E-10<br>1.89E-11<br>9.22E-15
	In lenti-HA-SPOP-Q165P groups:<br>DMSO (n = 15) vs. JQ1 (n = 5)<br>DMSO (n = 15) vs. CPI-637 (n = 5)<br>DMSO (n = 15) vs. JQ1 + CPI-637 (n = 5)<br>DMSO (n = 15) vs. NEO2734 (n = 5)<br>lenti-EV (DMSO, n = 15) vs. Lenti-HA-SPOP-Q165P (DMSO, n = 15)	 *<br>***<br>***<br>***<br>*	 0.049<br>1.74E-5<br>6.55E-13<br>1.01E-13<br>0.018
Fig 4E	Lenti-EV (DMSO, n = 4) vs. Lenti-EV (NEO2734, n = 4)<br>Lenti-EV (DMSO, n = 4) vs. Lenti-HA-SPOP-Q165P (DMSO, n = 4)<br>Lenti-HA-SPOP-Q165P (DMSO, n = 4) vs. Lenti-HA-SPOP-Q165P (NEO2734, n = 4)	**<br>*<br>**	0.008<br>0.047<br>0.003
Fig 4F	BRD4 enrichment at the enhancer of *c-MYC:*<br>DMSO (n = 3) vs. JQ1 (n = 3)<br>JQ1 + CPI-637 (n = 3) vs. NEO2734 (n = 3)	 **<br>n.s.	 0.0048<br>0.440
	BRD4 enrichment at the promoter of RAC1:<br>DMSO (n = 3) vs. JQ1 (n = 3)<br>JQ1 + CPI-637 (n = 3) vs. NEO2734 (n = 3)	 *<br>n.s.	 0.012<br>0.305
Fig 4G	Ac-H3 level at the enhancer of *c-MYC*:<br>DMSO (n = 3) vs. CPI-637 (n = 3)<br>JQ1 + CPI-637 (n = 3) vs. NEO2734 (n = 3)	 ***<br>***	 0.0067<br>0.0097
	Ac-H3 level at the promoter of *RAC1*:<br>DMSO (n = 3) vs. CPI-637 (n = 3)<br>JQ1 + CPI-637 (n = 3) vs. NEO2734 (n = 3)	 **<br>**	 0.0014<br>0.0020
Fig 5C	SPOP WT (Vehicle, n = 10) vs. Q165P (Vehicle, n = 10)<br>SPOP WT (Vehicle, n = 10) vs. SPOP WT (JQ1, n = 10)<br>SPOP Q165P (Vehicle, n = 10) vs. SPOP Q165P (JQ1, n = 10)<br>SPOP Q165P (JQ1, n = 10) vs. SPOP Q165P (NEO2734, n = 7)<br>SPOP Q165P (CPI-637, n = 9) vs. SPOP Q165P (NEO2734, n = 7)	***<br>***<br>***<br>***<br>***	1.69E-05<br>9.44E-09<br>2.10E-09<br>2.17E-05<br>2.53E-08
Fig 6D	BM1 (DMSO, n = 49) vs. BM1 (JQ1, n = 48)<br>BM1 (JQ1 + CPI-637, n = 52) vs. BM1 (NEO2734, n = 42)<br>BM5 (DMSO, n = 52) vs. BM5 (JQ1, n = 50)<br>BM5 (JQ1 + CPI-637, n = 49) vs. BM5 (NEO2734, n = 43)<br>ST1 (DMSO, n = 52) vs. ST1 (JQ1, n = 45)<br>ST1 (JQ1 + CPI-637, n = 52) vs. ST1 (NEO2734, n = 42)<br>313HR (DMSO, n = 50) vs. 313HR (JQ1, n = 48)<br>313HR (JQ1 + CPI-637, n = 50) vs. 313HR (NEO2734, n = 44)<br>ASC1 (DMSO, n = 50) vs. ASC1 (JQ1, n = 52)<br>ASC1 (JQ1 + CPI637, n = 50) vs. ASC1 (NEO2734, n = 38)<br>573R (DMSO, n = 52) vs. 573R (JQ1, n = 52)<br>573R (JQ1 + CPI-637, n = 53) vs. 573R (NEO2734, n = 45)	***<br>**<br>***<br>*<br>*<br>**<br>***<br>***<br>n.s.<br>***<br>*<br>***	0.00012<br>0.0049<br>1.21E-07<br>0.044<br>0.019<br>0.0014<br>9.54E-07<br>3.24E-11<br>0.699<br>6.11E-07<br>0.022<br>5.43E-17

Figure	Compared groups	Symbol	*P* Value
Fig 6F	SPOP WT groups (313HR):<br>DMSO (n = 10) vs. JQ1 (n = 10)<br>DMSO (n = 10) vs. CPI-637 (n = 10)<br>DMSO (n = 10) vs. JQ1 + CPI-637 (n = 10)<br>DMSO (n = 10) vs. NEO2734 (n = 10)	 ***<br>***<br>***<br>***	 6.88E-07<br>0.0006<br>7.79E-07<br>2.39E-10
	SPOP MUT groups (573R):<br>DMSO (n = 10) vs. JQ1 (n = 10)<br>DMSO (n = 10) vs. CPI-637 (n = 10)<br>DMSO (n = 10) vs. JQ1 + CPI-637 (n = 10)<br>DMSO (n = 10) vs. NEO2734 (n = 10)	 *<br>***<br>***<br>***	 0.025<br>4.32E-06<br>3.07E-09<br>7.31E-08
Fig 7B	EV (DMSO, n = 7) vs. EV (JQ1, n = 7)<br>EV (JQ1 + CPI-637, n = 7) vs. EV (NEO2734, n = 7)<br>Q165P (DMSO, n = 7) vs. Q165P (JQ1, n = 7)<br>Q165P (JQ1 + CPI-637, n = 7) vs. Q165P (NEO2734, n = 7)<br>F133V (DMSO, n = 7) vs. F133V (JQ1, n = 7)<br>F133V (JQ1 + CPI-637, n = 7) vs. F133V (NEO2734, n = 7)<br>EV (DMSO, n = 7) vs. Q165P (DMSO, n = 7)<br>EV (DMSO, n = 7) vs. F133V (DMSO, n = 7)	***<br>***<br>*<br>***<br>n.s.<br>***<br>***<br>***	2.13E-08<br>6.92E-07<br>0.015<br>3.85E-07<br>0.055<br>1.40E-13<br>0.0002<br>1.78E-06
Fig 7E	EV (DMSO, n = 4) vs. EV (NEO2734, n = 4)<br>Q165P (DMSO, n = 4) vs. Q165P (NEO2734, n = 4)<br>F133V (DMSO, n = 4) vs. F133V (NEO2734, n = 4)<br>EV (DMSO, n = 4) vs. Q165P (DMSO, n = 4)<br>EV (DMSO, n = 4) vs. F133V (DMSO, n = 4)	***<br>***<br>***<br>*<br>*	6.12E-05<br>5.52-05<br>4.71-05<br>0.044<br>0.032
Fig 7H	EV (Vehicle, n = 6) vs. EV (JQ1, n = 6)<br>Q165P (Vehicle, n = 6) vs. Q165P (JQ1, n = 6)<br>F133V (Vehicle, n = 6) vs. F133V (JQ1, n = 6)<br>EV (Vehicle, n = 6) vs. EV (NEO2734, n = 6)<br>Q165P (Vehicle, n = 6) vs. Q165P (NEO2734, n = 6)<br>F133V (Vehicle, n = 6) vs. F133V (NEO2734, n = 6)	***<br>**<br>*<br>**<br>***<br>***	1.90E-05<br>0.005<br>0.017<br>0.0047<br>1.65E-06<br>7.13E-07
Fig EV2A	BRD4 enrichment at the enhancer of *c-MYC*:<br>DMSO (n = 3) vs. JQ1 (n = 3)<br>JQ1 + CPI-637 (n = 3) vs. NEO2734 (n = 3)	 **<br>n.s.	 0.0017<br>0.416
	BRD4 enrichment at the promoter of *RAC1*:<br>DMSO (n = 3) vs. JQ1 (n = 3)<br>JQ1 + CPI-637 (n = 3) vs. NEO2734 (n = 3)	 **<br>n.s.	 0.0021<br>0.263
Fig EV2B	Ac-H3 level at the enhancer of *c-MYC*:<br>DMSO (n = 3) vs. CPI-637 (n = 3)<br>JQ1 + CPI-637 (n = 3) vs. NEO2734 (n = 3)	 **<br>***	 0.0093<br>3.05E-05
	Ac-H3 level at the promoter of *RAC1*:<br>DMSO (n = 3) vs. CPI-637 (n = 3)<br>JQ1 + CPI-637 (n = 3) vs. NEO2734 (n = 3)	 **<br>***	 0.0018<br>0.0002
Fig EV4B	313HR (WT, n = 12) vs. 573R (MUT, n = 14)	n.s.	0.133
Fig EV4D	313HR (WT, n = 3) vs. 573R (MUT, n = 3)	n.s.	0.445
Fig EV5D	SPOP WT (n = 60) vs. SPOP Q165P (n = 52)	**	0.0013
Fig EV5F	313HR (DMSO, n = 10) vs. 573R (DMSO, n = 10)<br>All 313HR treatment groups (n = 10)<br>All 573R treatment groups (n = 10)	*<br>n.s.<br>n.s.	0.0412<br>0.855<br>0.296
