# Supplementary material for: The novel BET‐CBP/p300 dual inhibitor NEO2734 is active in SPOP mutant and wild‐type prostate cancer
Source: EMBO Mol Med. 2019 Sep 26;11(11):e10659. doi: 10.15252/emmm.201910659 (PMC6835201; doi:10.15252/emmm.201910659)
Supplement: Supplementary file 2 — Expanded View Figures PDF [file EMMM-11-e10659-s002.pdf]

Expanded View Figures

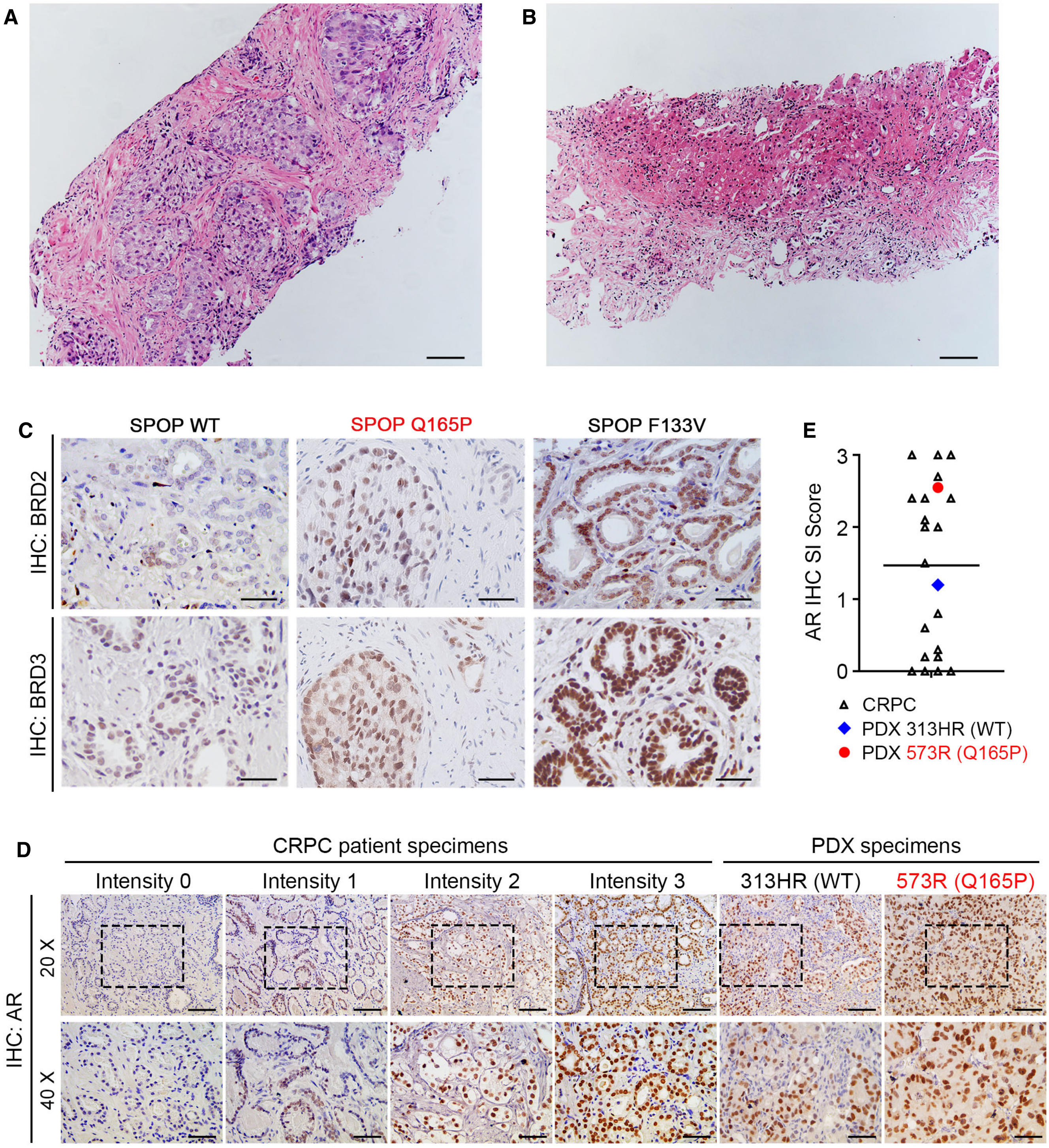

Figure EV1.

 © 2019 The Authors

Figure EV1. Histopathology images of a primary prostate tumor and a metastasis in liver harboring a heterozygous and homozygous SPOP Q165P mutation, respectively.

A, B Hematoxylin and eosin (H&E) staining was performed to determine pathologic diagnosis of a primary prostate cancer (A) and a metastatic lesion (B). Scale bars: 100 μm.

C IHC analysis of BRD2 and BRD3 protein expression in SPOP WT and MUT PCa patient samples (Q165P and F133V). Scale bars: 50 μm.

D, E IHC was conducted to examine AR protein expression in CRPC patient samples and two PDX samples including one WT and one Q165P. The representative images are shown in (D) with quantification data of IHC score in (E); CRPC samples (n = 20); PDX WT (n = 1); PDX Q165P (n = 1). The horizontal bar represents the mean. Scale bars: 100 μm for 20× fields; 50 μm for 40× fields.

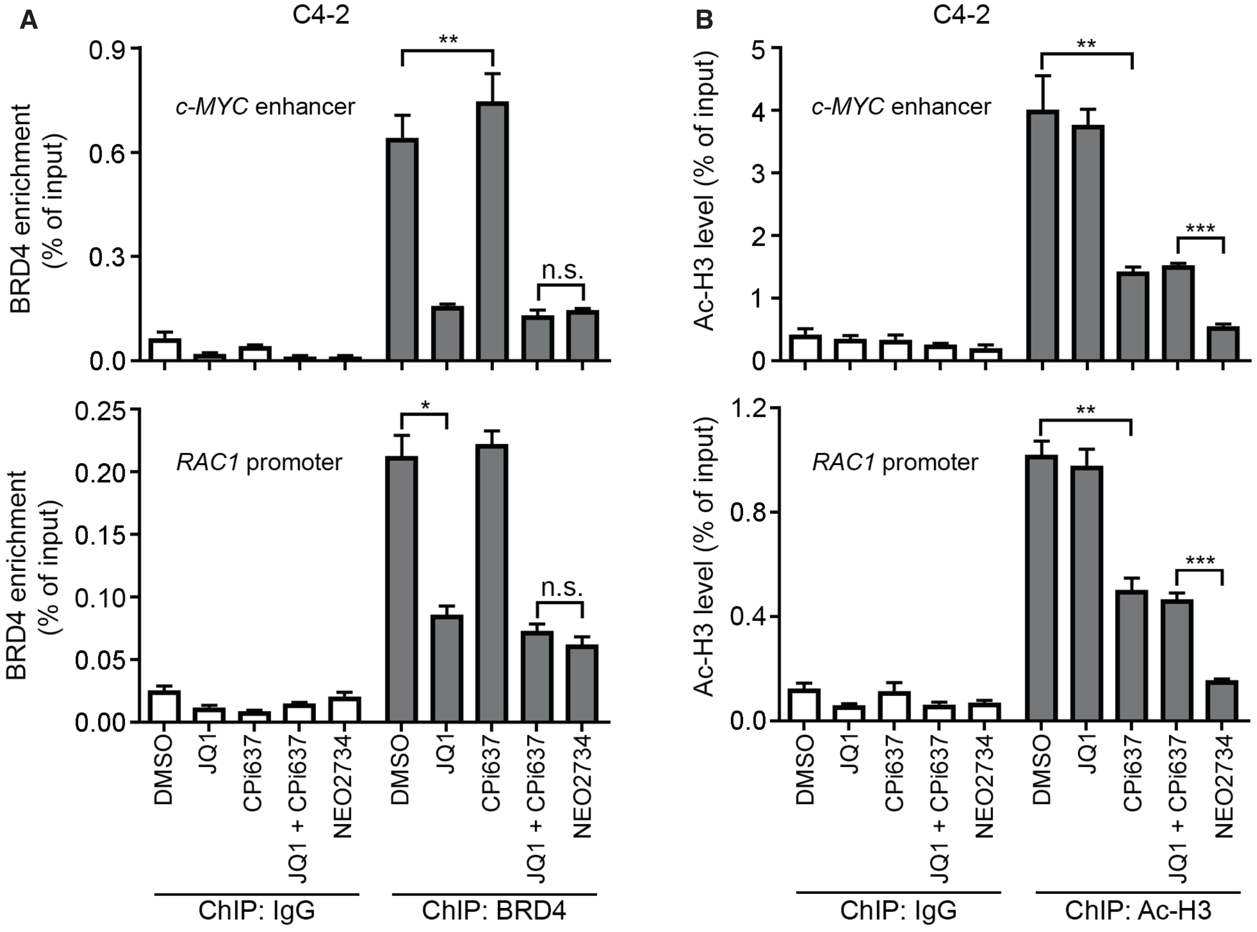

Figure EV2. NEO2734 is more superior in the inhibition of CBP/p300 BET domain than CPI-637.

A C4-2 cells were treated with the indicated inhibitors and harvested for ChIP with BRD4 antibody. The enrichment of BRD4 at *c-MYC* enhancer and *RAC1* promoter is determined by qPCR. All data shown are means ± SEM, n = 3. The P value was calculated by the unpaired two-tailed Student's t-test; n.s., not significant, *$P < 0.05$, **$P < 0.01$. See Appendix Table S4 for the detailed comparison, P Values.

B C4-2 cells were treated with the indicated inhibitors and harvested for ChIP with Ac-H3 antibody. The level of Ac-H3 at *c-MYC* enhancer and *RAC1* promoter is determined by qPCR. All data shown are means ± SEM, n = 3. The P value was calculated by the unpaired two-tailed Student's t-test; **$P < 0.01$, ***$P < 0.001$. See Appendix Table S4 for the detailed comparison, P values and sample number (n).

© 2019 The Authors

Figure EV3. Examination of BET, AR, and phosphorylated AKT protein in Q165P PDX tissues.

A Sanger sequencing confirmed the mutation status of SPOP Q165P in PDX tumors.
B Western blot analysis of the indicated proteins in the tumors from two SPOP WT and one Q165P PDX models. Each model had two different tumor samples, namely T1 and T2.
C IHC analysis of the level of BET proteins in two SPOP WT and one Q165P PDX models. The rectangles in the images indicate the enlarged area. Scale bars: 100 μm for 10× fields; 25 μm for 40× fields.
D Representative IFC images of AR and p-AKT-S473 protein expression in two SPOP WT and one Q165P PDX tumors. AR and p-AKT-S473 were stained in green. E-cadherin was used to define the membrane (red) and DAPI for the nucleus (blue). Scale bars: 25 μm.

Source data are available online for this figure.

© 2019 The Authors

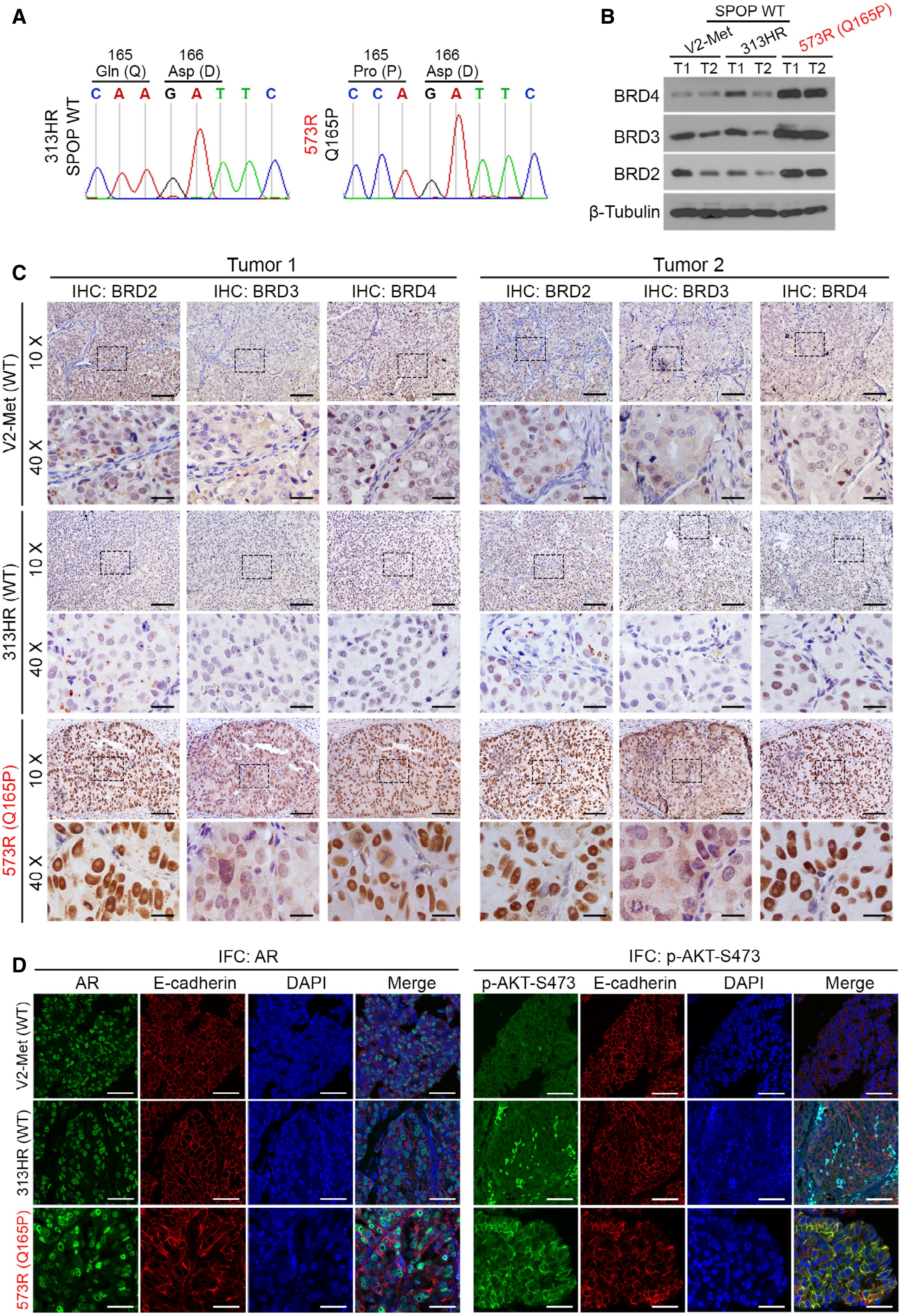
A
165
Gln (Q)
166
Asp (D)
C A A G A T T C
313HR
SPOP WT
165
Pro (P)
166
Asp (D)
C C A G A T T C
573R
Q165P
B
SPOP WT
V2-Met
313HR
573R (Q165P)
T1 T2 T1 T2 T1 T2
BRD4
BRD3
BRD2
β-Tubulin
C
Tumor 1
Tumor 2
IHC: BRD2
IHC: BRD3
IHC: BRD4
V2-Met (WT)
313HR (WT)
573R (Q165P)
10 X
40 X
D
IFC: AR
IFC: p-AKT-S473
AR
E-cadherin
DAPI
Merge
p-AKT-S473

Figure EV3.

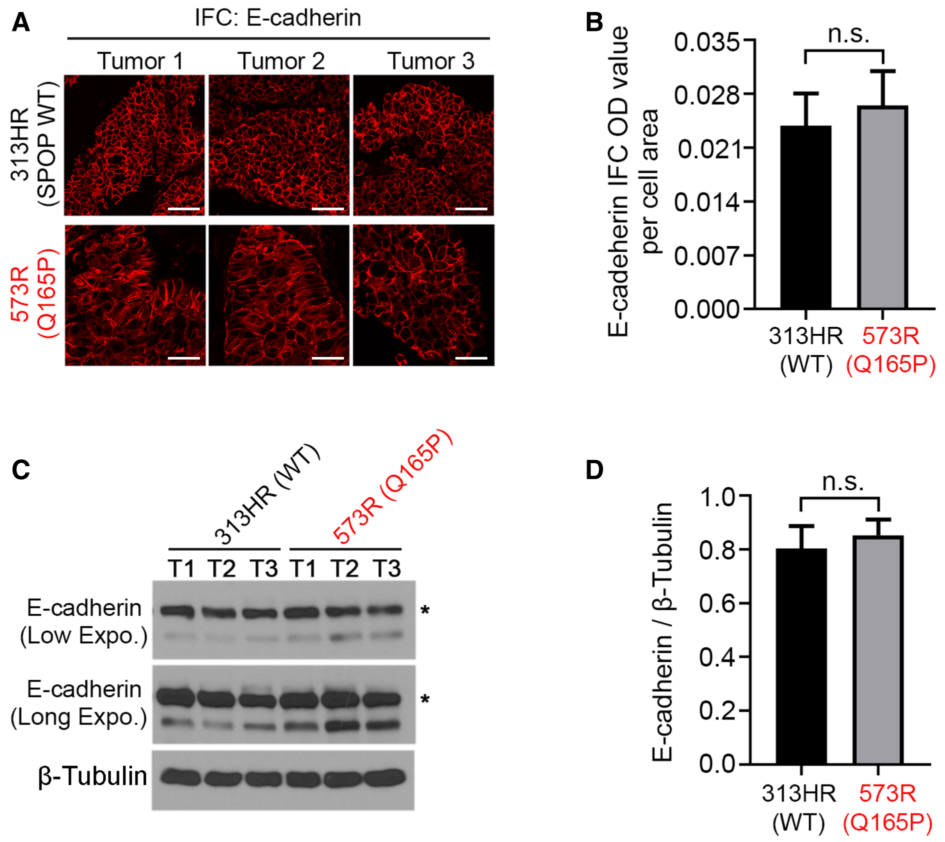

Figure EV4. SPOP mutation appears no effects on the expression of E-cadherin protein.

A, B The IFC representative images of E-cadherin staining in PDX tumors are shown in (A). ImageJ was used to quantify the optical density (OD)/area (pixel) in (B). All data shown are mean values ± SEM, n = 12 for WT and n = 14 for Q165P. The P value was calculated by the unpaired two-tailed Student's t-test; n.s., not significant. See Appendix Table S4 for the detailed comparison, P values and sample number (n). Scale bars: 25 μm.

C, D Western blot analysis was performed to determine the protein level in PDX tumors in (C) with the quantification data in (D). Each model had three different tumor samples, namely T1, T2, and T3. Asterisk, E-cadherin at expected molecular mass. All data shown are mean values ± SEM, n = 3. The P value was calculated by the unpaired two-tailed Student's t-test; n.s., not significant. See Appendix Table S4 for the detailed comparison, P values, and sample number (n).

Source data are available online for this figure.

Figure EV5. NEO2734 suppresses the growth of organoids not through inhibiting cell proliferation.

A A schematic shows the procedure of generation of organoids from PDX tumors. Scale bars: 50 μm.

B Sanger sequencing confirmed the mutation status of SPOP Q165P in organoids.

C, D The 313HR (SPOP WT) and 573R (Q165P) organoids were cultured for 10 days and photographed (C) and the organoid diameter was quantified (D). All data shown are box + whiskers (Min to Max), n = 60 for WT and n = 52 for Q165P. In the box plot, the horizontal lines represent median and the box ranges are between the 25th percentile and the 75th percentile. The P value was calculated by Wilcoxon rank-sum test with continuity correction; $^{**}P < 0.01$. See Appendix Table S4 for the detailed comparison, P values, and sample number (n). Scale bars: 50 μm.

E IFC of AR in six organoid lines including four SPOP WT (BM1, BM5, ST1, and 313HR) and two MUT (ASC1 and 573R). E-cadherin antibody was used to define the cell membrane (red) and DAPI for nucleus (blue). Scale bars: 25 μm.

F, G SPOP WT and Q165P organoids were cultured for 5 days, followed by the treatment with the indicated inhibitors for 5 days. The representative images from Ki67 IFC staining are shown in (E). Scale bars: 25 μm. The quantification data of the percentage of Ki67-positive cells in every organoid are shown as means ± SEM (F), n = 10. The P value between two groups was calculated by the unpaired two-tailed Student's t-test. The P value among several treatment groups was evaluated by one-way ANOVA, which is indicated by red line; n.s., not significant, $^{*}P < 0.05$. See Appendix Table S4 for the detailed comparison, P values, and sample number (n).

▶

 © 2019 The Authors

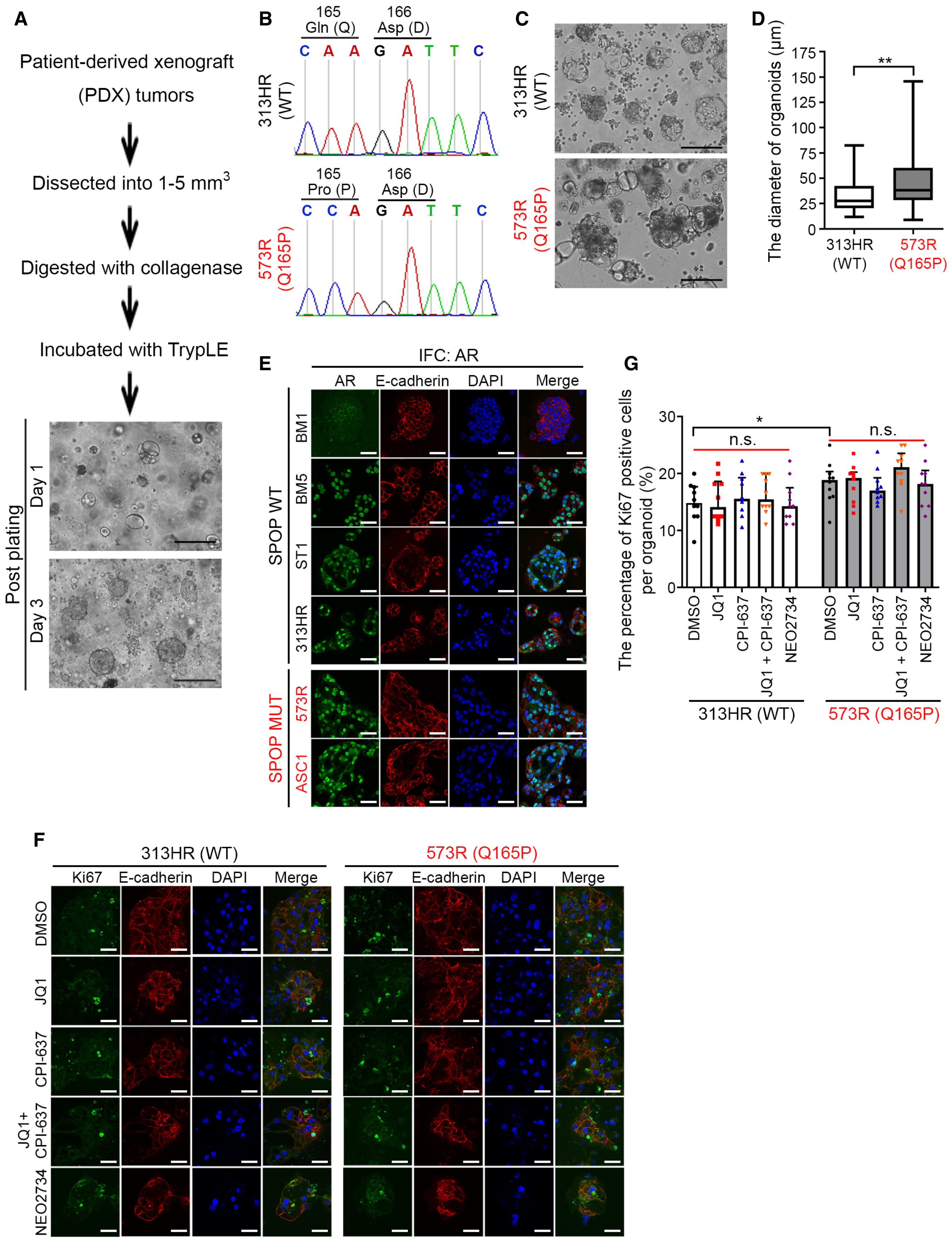
A
Patient-derived xenograft (PDX) tumors
Dissected into 1-5 mm3
Digested with collagenase
Incubated with TrypLE
Post plating
Day 1
Day 3
B
165 Gln (Q)
166 Asp (D)
C A A G A T T C
313HR (WT)
165 Pro (P)
166 Asp (D)
C C A G A T T C
573R (Q165P)
C
313HR (WT)
573R (Q165P)
D
The diameter of organoids (μm)
**
313HR (WT)
573R (Q165P)
E
IFC: AR
AR
E-cadherin
DAPI
Merge
SPOP WT
BM1
BM5
ST1
313HR
SPOP MUT
573R
ASC1
G
The percentage of Ki67 positive cells per organoid (%)
*
n.s.
n.s.
DMSO
JQ1
CPI-637
JQ1 + CPI-637
NEO2734
313HR (WT)
573R (Q165P)
F
313HR (WT)
573R (Q165P)
Ki67
E-cadherin
DAPI
Merge
DMSO
JQ1
CPI-637
JQ1+ CPI-637
NEO2734

Figure EV5.
