# Supplementary figures and images for: The novel BET‐CBP/p300 dual inhibitor NEO2734 is active in SPOP mutant and wild‐type prostate cancer
Source: EMBO Mol Med. 2019 Sep 26;11(11):e10659. doi: 10.15252/emmm.201910659 (PMC6835201; doi:10.15252/emmm.201910659)

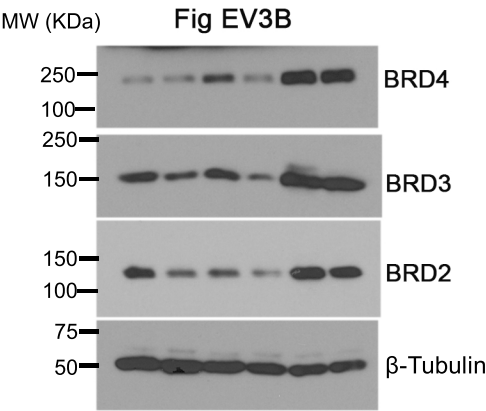

Supplement: Supplementary file 4 — Source Data for Expanded View [file EMMM-11-e10659-s010.zip › EMM-2019-10659-V3_SourceDataForFigureEV3B.pdf]

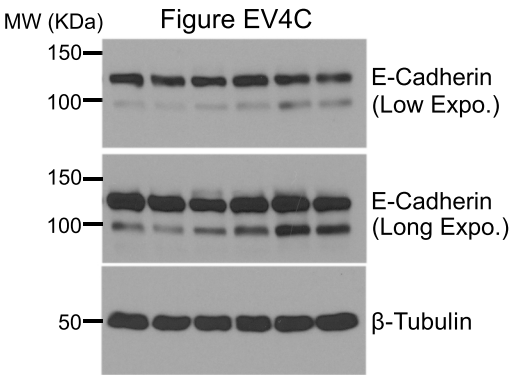

Supplement: Supplementary file 4 — Source Data for Expanded View [file EMMM-11-e10659-s010.zip › EMM-2019-10659-V3_SourceDataForFigureEV4C.pdf]

Figure 2 (Full unedited images)

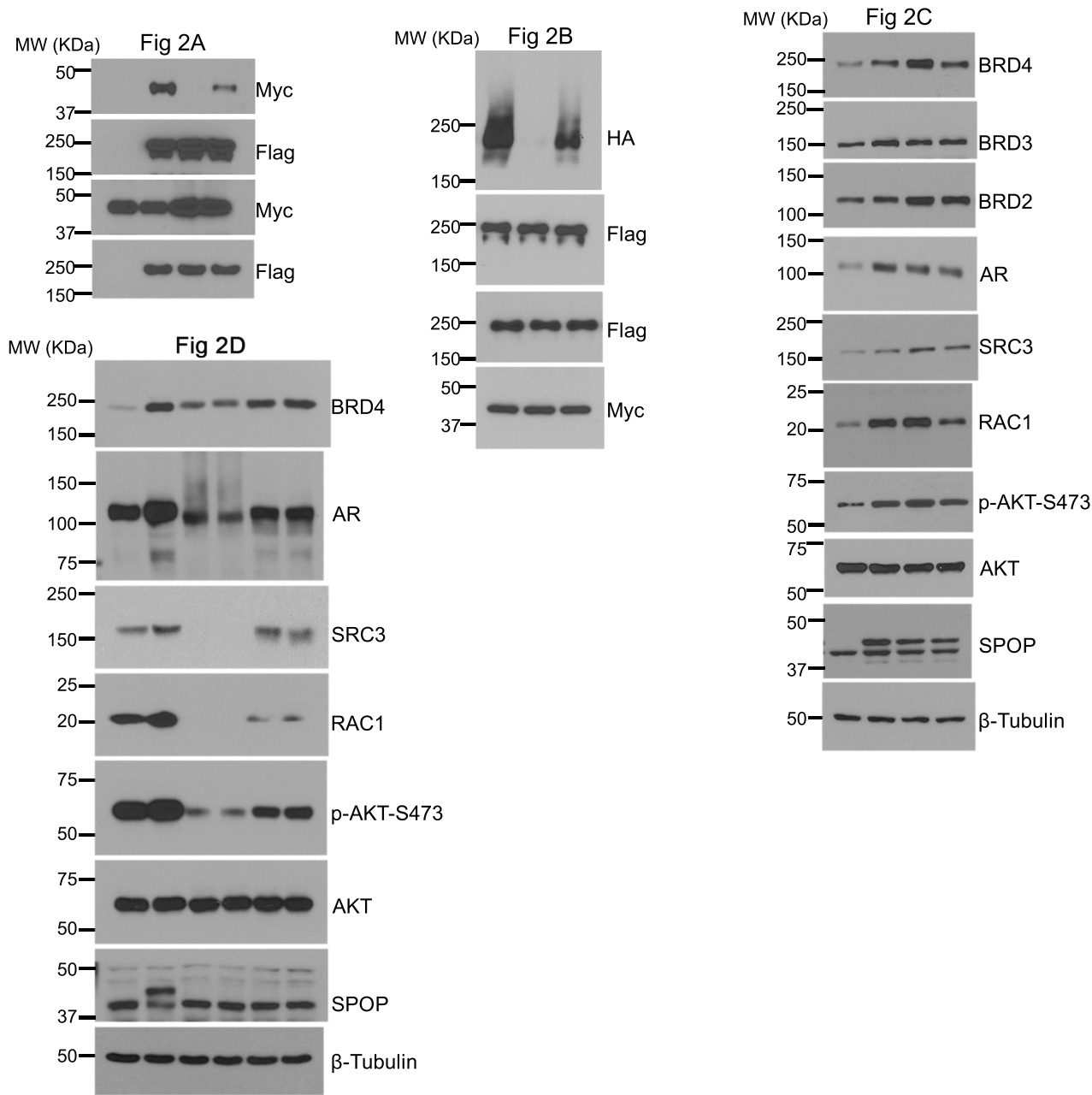

Supplement: Supplementary file 6 — Source Data for Figure 2 [file EMMM-11-e10659-s004.pdf]

Figure 3 (Full unedited images)

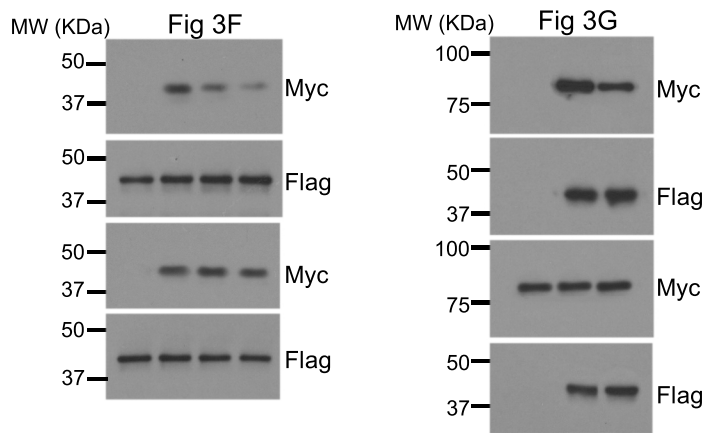

Supplement: Supplementary file 7 — Source Data for Figure 3 [file EMMM-11-e10659-s005.pdf]

Figure 4 (Full unedited images)

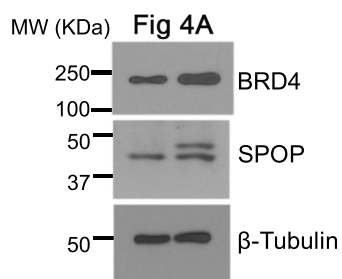

Supplement: Supplementary file 8 — Source Data for Figure 4 [file EMMM-11-e10659-s006.pdf]

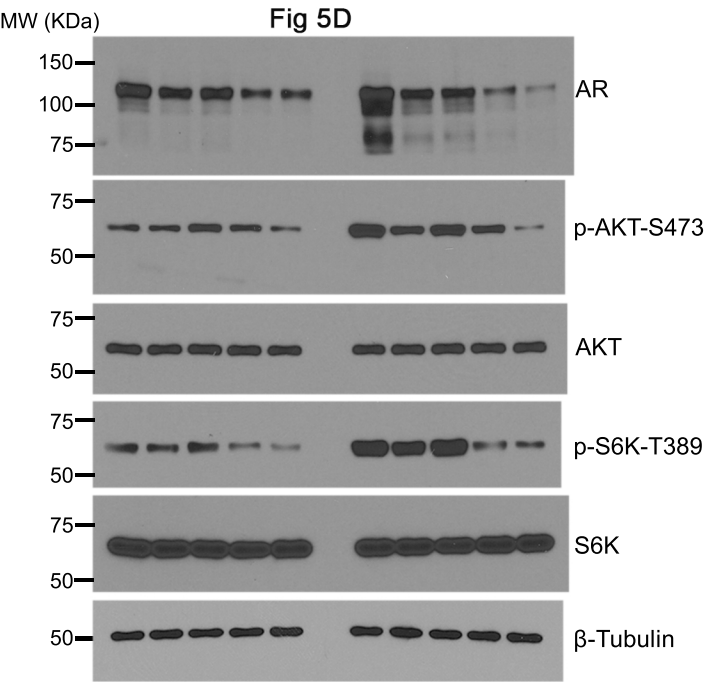

Supplement: Supplementary file 9 — Source Data for Figure 5 [file EMMM-11-e10659-s007.pdf]

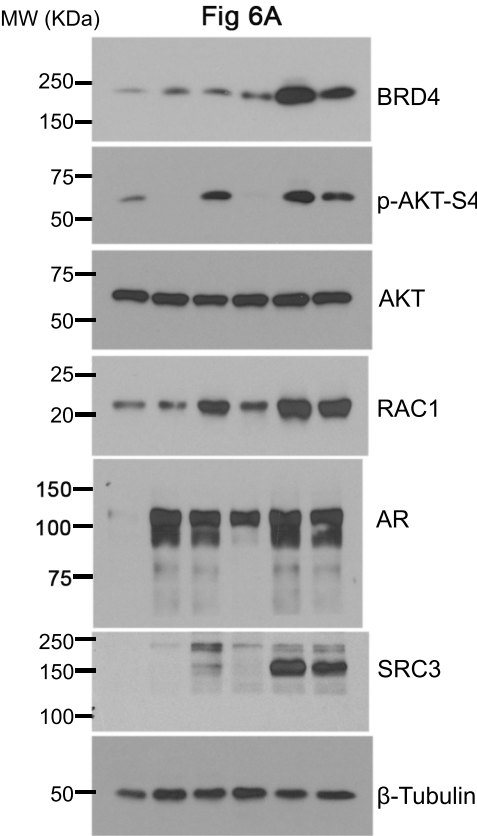

Supplement: Supplementary file 10 — Source Data for Figure 6 [file EMMM-11-e10659-s008.pdf]

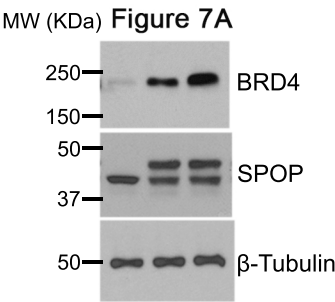

Supplement: Supplementary file 11 — Source Data for Figure 7 [file EMMM-11-e10659-s009.pdf]
